# Balancing Collective Exploration and Exploitation in Multi-Agent and Multi-Robot Systems: A Review

**DOI:** 10.3389/frobt.2021.771520

**Published:** 2022-02-01

**Authors:** Hian Lee Kwa, Jabez Leong Kit, Roland Bouffanais

**Affiliations:** ^1^ Singapore University of Technology and Design, Singapore, Singapore; ^2^ Thales Solutions Asia, Singapore, Singapore; ^3^ University of Ottawa, Ottawa, ON, Canada

**Keywords:** dynamic environment, exploration, exploitation, multi-agent systems, multi-robot systems, swarm intelligence, swarm robotics

## Abstract

Multi-agent systems and multi-robot systems have been recognized as unique solutions to complex dynamic tasks distributed in space. Their effectiveness in accomplishing these tasks rests upon the design of cooperative control strategies, which is acknowledged to be challenging and nontrivial. In particular, the effectiveness of these strategies has been shown to be related to the so-called exploration–exploitation dilemma: i.e., the existence of a distinct balance between exploitative actions and exploratory ones while the system is operating. Recent results point to the need for a dynamic exploration–exploitation balance to unlock high levels of flexibility, adaptivity, and swarm intelligence. This important point is especially apparent when dealing with fast-changing environments. Problems involving dynamic environments have been dealt with by different scientific communities using theory, simulations, as well as large-scale experiments. Such results spread across a range of disciplines can hinder one’s ability to understand and manage the intricacies of the exploration–exploitation challenge. In this review, we summarize and categorize the methods used to control the level of exploration and exploitation carried out by an multi-agent systems. Lastly, we discuss the critical need for suitable metrics and benchmark problems to quantitatively assess and compare the levels of exploration and exploitation, as well as the overall performance of a system with a given cooperative control algorithm.

## 1 Introduction

In recent years, there has been an increasing interest in using multi-agent systems (MAS) to carry out a wide array of complex tasks. In using such collaborative platforms, a large and challenging task can be broken down into smaller tasks that can be tackled by agents with the ability to change their actions based on their local environment (Dorri et al., 2018). This provides MAS with very high levels of flexibility—i.e., adaptability to changing circumstances—thereby making them an attractive solution to problems in fields ranging from traffic management (Rehman et al., 2018), to machine learning (Madhushani and Leonard, 2020), and robotics (Quattrini Li, 2020; Schranz et al., 2021).

A well-known challenge faced by all MAS is the exploration–exploitation dilemma. This dilemma arises owing to the fact that gathering new information (i.e., exploration) and making use of currently available information (i.e., exploitation) tend to be two mutually exclusive activities (Berger-Tal et al., 2014). Should a system be setup in favor of exploration, it would be able to gather large amounts of knowledge without being fully able to benefit from this knowledge. This may increase the time required for the MAS to accomplish its task or prevent it from achieving its goal altogether (Dadgar et al., 2016). On the other hand, should a system be setup to favor exploitation, the system may be unable to adapt to changes in the environment or may find itself trapped in a local optimum (Azoulay-Schwartz et al., 2004; Berger-Tal et al., 2014; Mehlhorn et al., 2015). Despite this apparent trade-off between the two activities, both sets of actions can be performed simultaneously (Stadler et al., 2014). As such, one can expect that to maximize the performance of such a system, there ought to be some sort of balance—most likely a dynamic one—between the amount of exploration and exploitation carried out.

Within the field of robotics, MAS have been studied for their applications within Multi-Robot Systems (MRS) (Bayindir, 2016; Schranz et al., 2020; Dias et al., 2021). The use of MRS, which can either be controlled in a centralized or decentralized fashion (Khan et al., 2015), has been demonstrated in a wide variety of tasks, including area mapping (Okumura et al., 2018; Kit et al., 2019), area characterization (Ebert et al., 2018; Ebert et al., 2020), collective construction (Werfel et al., 2014), collective decision-making (Valentini et al., 2017), collective transport (Takahashi et al., 2014), perimeter defense or geofencing (Chamanbaz et al., 2017; Shishika and Paley, 2019), as well as target search and tracking (Kamimura and Ohira, 2010; Shah and Schwager, 2019; Kwa et al., 2020a). The attractiveness of MRS in such tasks stem from three key features: 1) flexibility—the ability for the to adapt quickly to rapidly changing environments, 2) robustness—the ability to cope with component failures within the system, and 3) scalability—the ability to carry out tasks in systems comprised of different number of agents (Dorigo et al., 2021).

For implementation of swarm strategies in MRS, agent behaviors are largely developed in simulation before being tested in physical systems. However, doing so inevitably leads to the so-called *reality gap*, in which there is a difference between the simulation models and the actual robots used (Francesca and Birattari, 2016; Mouret and Chatzilygeroudis, 2017). This could manifest as differences between the actual robot behaviors and those predicted by simulations due to various factors such as variations in robot kinematics, communications noise, and other environmental factors affecting the robot’s movements. Given the potentially large number of agents involved, this reality gap may be further widened when modeling MRS. While this indeed poses a unique problem for MRS practitioners, in this paper, we only focus on the behavioral aspects of how robotic agents balance their efforts in exploration and exploitation.

At this point, it should be noted that there is a marked difference in the strategies that are employed for use in static and dynamic tasks. While there is still the need to balance exploration and exploitation when operating in static environments, this balance tilts heavily in favor of exploitation, especially during the latter stages of the task. For example, in the search for a static target, Yang et al. (2019) performed an initial random search before transitioning to a Particle Swarm Optimization (PSO) algorithm to facilitate the convergence of agents around the target’s position. Similarly, Matignon and Simonin (2018) found that biasing the swarm towards exploitation during the latter phases of a static target search task greatly reduces the time taken to accomplish the given mission. Tasks in slow-evolving environments can also be treated as a quasi-static problem and be completed in the same manner. Coquet et al. (2019) were able to demonstrate a simulated swarm using a comparable algorithm to search for and track a slow-moving target. Due to the focus on exploitation during the latter stages of the tasks, research on MAS carrying out static or quasi-static tasks tend to focus on convergence time and the accuracy of the overall system (Zou et al., 2015; Ebert et al., 2018; Ebert et al., 2020).

Given this critical distinction between static, quasi-static, and fast-evolving tasks, we propose the following definition for the latter: a task occurring in an environment that evolves at a rate at which a single agent is unable to keep up. Examples include tracking a target that can move faster than the agents (Janosov et al., 2017; Kwa et al., 2020a), and dynamic features that lead to a notable evolution in the optimum agent allocation of a task assignment problem (Kazakova et al., 2020). For a system to effectively carry out its assigned task in a fast-moving dynamic environment, there must be some form of adjustment of the balance between exploratory and exploitative actions throughout the duration of the task. Should an MAS bias its actions towards exploitation over time, such exploitative activity based on outdated information will inevitably result in poorer system performances (Jordehi, 2014; Pitonakova et al., 2018; Phan et al., 2020). Despite this, there is still merit in studying the methods used to control the level of exploration and exploitation carried out in MAS operating in static and quasi-static environments. This is because such systems still are susceptible to being trapped in local optima during the initial stages of the task (Leonard et al., 2011; Zou et al., 2015; Ghassemi and Chowdhury, 2019) and variations of strategies employed in static environments have been modified and applied for use in fast-evolving environments (Senanayake et al., 2016).

**FIGURE 1:**
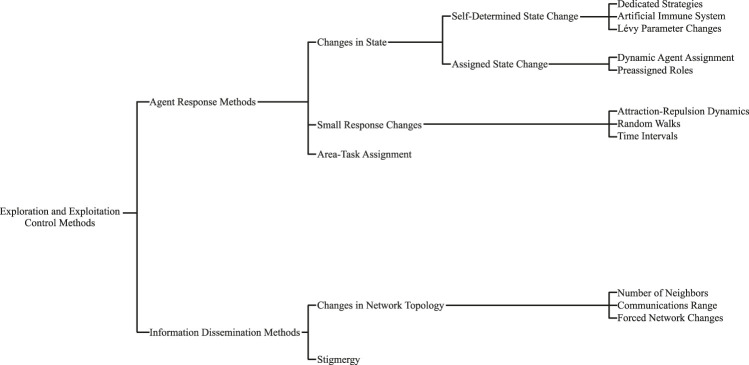
Classification of MAS exploration and exploitation control methods.

Given the notable importance of the exploration vs. exploitation balance in MAS, we present here a survey of the methods and techniques used to control this balance. First, we explore the concept of exploration and exploitation and the common metrics that practitioners use to quantify the level of exploration and exploitation carried out by their systems. Next, we go through the methods used to control the exploration and exploitation balance. An overview of the types of methods and strategies explored within this review can be found in Figure 1. In the conclusion, we present possible directions for future research as well as summarizing remarks.

## 2 Exploration and Exploitation

### 2.1 Characterizing Exploration and Exploitation

Exploration and exploitation is a *trans*-disciplinary concept used in a variety of fields and contexts. As such, there is no unique definition that can be used to define these two behaviors. Nevertheless, these exploratory and exploitative actions can be characterized by certain observable features. To this end Mehlhorn et al. (2015), identifies three key distinct features that distinguish between the two: 1) behavioral patterns, 2) the uncertainties associated with an actor’s choices, and 3) the expected outcomes as a result of an agent’s actions. It is important to note at this point that while these three features classify the actions of a single agent, a single agent’s actions do not characterize the exploration–exploitation dynamics (EED) of the entire MAS. It is only through observing the actions of all or a proportion of a system’s agents that one is able to accurately characterize the exploration–exploitation balance of an MAS.

An agent’s behavioral patterns are one of the most common definitions of exploration and exploitation used as the movements it carries out is a physical quantity that can be directly observed and measured. Many MAS practitioners use the agents’ movements and observed positions to determine the system’s exploration-exploitation balance (see Section 2.2.1). For example, in a target search context, when agents are close together or traveling towards the same region of the area of operations, it can be assumed that they are exploiting a common source of information (Jordehi, 2014; Kwa et al., 2021b). Conversely, if agents are moving away from each other or in a random fashion, they can be assumed to be carrying out exploration.

The less tangible aspect of an agent’s behavioral patterns are its intentions that drive the observed actions. While not as obvious as an agent’s motions, these can still be easily determined by querying the state of an individual agent in an engineered MAS. In their review, Stadler et al. (2014) posited that exploration and exploitation are associated with a certain level of learning. Therefore it can be said that an agent is carrying out exploitation if it uses previously acquired knowledge and carrying out exploration if it is not doing so (Gupta et al., 2006; Nauta et al., 2020b). Again, this is another common method of defining exploration and exploitation in MAS, where certain practitioners have developed dedicated behaviors that facilitate exploration and exploitation separately (see Section 3.1.1.1).

Besides observing an agent’s behavioral patterns, the amount of uncertainty associated with each action can also be used to classify exploration and exploitation. With this characteristic, exploratory actions are associated with those that have high levels of uncertainty, while exploitative ones are associated with low levels of uncertainty. In MAS operating in unknown environments, these uncertainties are usually presented in the form of a map that updates itself as the task progresses (Wolek et al., 2020; Crosscombe and Lawry, 2021; Liu et al., 2021). Using such maps allows an agent to move towards regions of different levels of uncertainty based on its intentions to carry out either exploration or exploitation. As such, an agent that moves towards or gathers information in an area with high uncertainty is deemed to be carrying out exploration, while an agent doing the same in an region of low uncertainty can be said to be carrying out exploitation.

Finally, the type of action an agent performs can be classified based on the expected results of the said action. This is typically exemplified by observe-or-bet tasks. In such a task, an agent is given a chance to observe the environment and gain information while not receiving any reward (exploration). Alternatively, the agent may “bet” on an option and receive its associated reward (exploitation), although the amount of reward received remains hidden from the agent (Tversky and Edwards, 1966; Navarro et al., 2016). While these tasks offer a very clear distinction between exploration and exploitation, such contrasts are not as explicit when observing real systems; agents tend to know the quantity of a reward and the action that was performed that led to the reward. These additional pieces of knowledge allow an agent to obtain more information about its surrounding environment and allow agents to simultaneously perform exploration and exploitation (Gupta et al., 2006).

As highlighted by Mehlhorn et al. (2015), these three characteristics need not be used to define exploration and exploitation in isolation. Multiple characteristics can be used to determine the type of activity being carried out by an agent. For example, when performing an area mapping task, Matignon and Simonin (2018) proposed using an MRS that built an occupancy grid map that increased in resolution as the task progressed. If a robot chose to move into a previously unvisited cell (high uncertainty, learning expected), it was determined to be exploring. If the robot chose to move to a cell with a known object of interest or update its map of the local area with a higher resolution (low uncertainty, reward expected), it was determined to be exploiting.

### 2.2 Measuring Exploration and Exploitation

After selecting their definitions of exploration and exploitation, practitioners next turn their attention to measuring the level and quantifying the balance between the two activities. In the field of computational optimization, a typical strategy in finding a global optimum is through the use of an MAS consisting of many candidate solutions searching the solution space. In this use case, the quantification and monitoring of the system’s EED is important as doing so permits it to autonomously detect if the solutions have been trapped in a local optimum or have stagnated. Doing so grants the system the ability to self-modify specified parameters, allowing it to “jump out” and continue with its optimization task (Palmieri and Marano, 2016; Bonyadi and Michalewicz, 2017). Similarly in MAS operating in dynamic environments, these measurements also allow a system to monitor and prevent solution diversity loss, allowing systems to track moving optima over time (Blackwell and Branke, 2006; Leonard et al., 2011; Jordehi, 2014). At this point, it should be noted that a system’s diversity is analogous to the amount of exploration it carries out. These metrics mostly fall within the categories of spatial distribution based metrics or probability based metrics, although there are also other metrics that have been used that do not fall within these two categories.

While most exploration and exploitation metrics are measured globally, i.e., a system-wide property that allows one to quantify a system’s EED as a whole, some of these metrics can also be measured locally by the individual agents, thereby allowing them to adjust their actions appropriately. The use of globally and locally computed metrics both have their associated advantages and disadvantages. While the use of a system-wide EED metric would allow for a more accurate quantification of the overall balance between exploration and exploitation, it also entails that this balance be controlled in a centralized fashion. This would result in the loss of a certain degree of system scalability, flexibility, and robustness. Conversely, if a locally computed metric is used, the agents would be able to use this metric during their operations, allowing them to influence the behavior of the system through various feedback or feedforward loops. While such agent-based metrics may not be able to quantify a system’s EED as accurately as a global metric, it allows the system to maintain a level of decentralization and hence, scalability, flexibility, and robustness.

#### 2.2.1 Spatial Distribution Based Metrics

Similar to classifying an agent’s actions based on its behavior and movement patterns, the use of metrics based on the agents’ spatial distribution is very popular as it is a physical quantity that can be easily observed, measured, and calculated. As previously mentioned, to quantify the level of exploration and exploitation in computational MAS, the solution diversity of a system is usually calculated. This solution diversity is a measure of the spatial distribution of all candidate solutions within the feasible region. Cheng et al. (2015) based their diversity metric on the average distance of each solution from the mean. Hussain et al. (2019) adapted this metric for use in their study of swarm-based metaheuristic algorithms by using the median solution instead of the mean as it reflected the population center of the candidate solutions more accurately. In a similar fashion Zhan et al. (2009), calculated a *population standard deviation* to measure the solution spread. In summary, all these metrics are essentially based on low-order indicators of the statistical distribution.

Comparable metrics are in use to measure the amount of exploration and exploitation carried out by agents within MRS. As seen with the metrics used in computational optimization, measuring the spatial distribution of agents is also used to quantify the level of exploration and exploitation. In their target search task Dadgar et al. (2017) and Dadgar et al. (2020), measured the diversity of individual robots within their MRS by calculating the amount of overlap between a robot and its neighbors’ search areas. Agent sensor area overlap was also used by Sardinha et al. (2020a) who calculated the size of area covered by at least two agents as a measure of the amount of effective exploration performed by their swarm in a surveillance task. This metric is similar to the one used by Esterle and Lewis (2017), Esterle and Lewis (2020) and Frasheri et al. (2020) who used a *k*-coverage metric that calculated the amount of time a target spent being tracked by at least *k* agents.

Stogiannos et al. (2020) measured the level of exploration and exploitation by tracking three metrics: 1) the minimum distance between agent pairs, 2) the sum of the distances between detected targets and their closest agents, and 3) the distance moved by each agent over the course of one time-step. While often not explicitly stated, the distance between a robot and its neighbors is the most common measure of diversity as most control methods tend to focus on preventing excessive aggregation or spatial distribution (Hereford and Siebold, 2010; Meyer-Nieberg et al., 2013; Lv et al., 2016; Meyer-Nieberg, 2017; Chen and Huang, 2019; Coquet et al., 2019; Coquet et al., 2021).

The lack of distance measurements does not preclude the use of spatial distribution metrics. As a proxy for the amount of exploitation performed by each robot Yang et al. (2019), calculated a communications-based aggregation degree from the number of neighbors within visual range, with higher numbers of agents within visual range suggesting a higher amount of aggregation. This eliminates the need to share positional information between agents, allowing an agent to reduce the number of radio transmissions it needs to make. In an adaptation to the BEECLUST algorithm, which was first proposed by Kernbach et al. (2009), Schmickl et al. (2009) and Wahby et al. (2019) determined the level of local aggregation, and therefore the level of exploitation of individual agents, by measuring the time interval between two consecutive neighboring robot encounters.

The aggregation degree may not necessarily need to be calculated with the use of spatial information. In performing a search for multiple targets using several sub-swarms comprised of leaders and followers Tang et al. (2020a), calculate the aggregation of the sub-swarms by comparing the fitness value of each sub-swarm leader to the mean fitness value of all the leaders within the swarm. This is similar to the aggregation degree proposed by Yang et al. (2007), who calculated the ratio between the agent with the lowest fitness value and the agent with the highest fitness value. A list of works using spatial metrics to quantify an MAS’ EED can be found in Table 1.

**TABLE 1:** List of works using spatial distribution metrics to quantify MAS exploration and exploitation.

References	Metric	Task
Sardinha et al. (2020a)	Area observed by at least two agents	Area Coverage
Blackwell and Branke (2006), Hereford and Siebold (2010), Leonard et al. (2011), Jordehi (2014)	Euclidean distance between solutions	Optimization
Cheng et al. (2015)	Euclidean distance between solution and mean system solution	Optimization
Hussain et al. (2019)	Euclidean distance between solution and median system solution	Optimization
Zhan et al. (2009)	Standard deviation of all candidate solutions	Optimization
Tang et al. (2020a)	Fitness value between sub-swarm leaders and mean fitness value	Optimization
Schmickl et al. (2009), Wahby et al. (2019)	Local agent density	Optimal Area Aggregation
Palmieri and Marano (2016), Dadgar et al. (2016), Dadgar et al. (2017), Dadgar et al. (2020)	Agent sensor overlap region	Target Search
Meyer-Nieberg et al. (2013), Lv et al. (2016), Meyer-Nieberg (2017), Chen and Huang (2019), Coquet et al. (2019), Coquet et al. (2021)	Euclidean distance between agents	Target Search
Yang et al. (2019)	Number of agents within visual range	Target Search
Esterle and Lewis (2017), Esterle and Lewis (2020), Frasheri et al. (2020)	*k*-Coverage	Target Tracking
Stogiannos et al. (2020)	Distance between agents, distance between targets and agents & distance moved by agents in one time-step	Target Tracking

#### 2.2.2 Probability Based Metrics

While less popular than the use of spatial distance metrics, the probability of actions carried out by individual agents can also be used to quantify the level of exploration and exploitation involved. The most common use of probability based metrics can be found in the application of Lévy walks and flights. Lévy walks are a class of random walks where the distance of the walk, *l*, is taken from a heavy-tailed probability distribution function, *P*(*l*). This probability distribution function is given by the equation:


$$P\left(l\right)∼l^{-\mu },$$ (1)

where *μ* is the Lévy parameter controlling the shape of the probability distribution function. Lower values of *μ* close to unity lead to a higher probability of longer walk lengths between direction changes, which is associated with higher levels of exploration. Conversely, higher values of *μ* cause an agent to preferentially carry out shorter walks between changes of direction, characteristic of higher levels of exploitation (Viswanathan et al., 2000; Viswanathan et al., 2008). The use of the Lévy parameter has been used as a method of quantifying and controlling the level of exploration and exploitation of various MRS applied in different tasks, ranging from foraging (Zedadra et al., 2019; Nauta et al., 2020a; Nauta et al., 2020b; Nauta et al., 2020c), to target search (Senanayake et al., 2016; Harikumar et al., 2019; Pang et al., 2019), to area mapping (Ramachandran, 2018; Kegeleirs et al., 2019; Ramachandran et al., 2020).

In addition to the use of Lévy distributions, action probabilities can be used to set the level of exploration and exploitation carried out by an MRS. To search for a target Matignon and Simonin (2018), used action probabilities to bias an agent in favor of carrying out either exploratory or exploitative actions. In a similar fashion, Falcón-Cortés et al. (2019), Nauta et al. (2020a) and Nauta et al. (2020b) uses the probability of an agent using information in its memory as means of determining and controlling the level of exploration and exploitation of the system. In their best-of-*n* problem Prasetyo et al. (2019), used an opinion switching probability threshold as a proxy to measure and control the exploration and exploitation dynamics of their swarming system. With this, higher switching probabilities are associated with exploration, while lower probabilities are associated with exploitation. These probability thresholds are also popular in the related field of task allocation to obtain the optimal distribution of agents among all the tasks presented to the MRS. In such scenarios, the use of probability thresholds allows an agent to decide whether it should stay and continue with its current task (exploitation) or move on and attempt to perform another task within the environment (exploration) (de Lope et al., 2015; Lee and Kim, 2019; Kazakova et al., 2020; Lee et al., 2020). A list of works using such probability based metrics can be found in Table 2 for different tasks.

**TABLE 2:** List of works using probability based metrics to quantify MAS exploration exploitation dynamics.

References	Metric	Task
Ramachandran (2018), Kegeleirs et al. (2019), Ramachandran et al. (2020)	Lévy parameter	Area Mapping
Prasetyo et al. (2018), Prasetyo et al. (2019)	Opinion switching probability	Best-of-*n* Problem
Zedadra et al. (2019), Nauta et al. (2020c)	Lévy parameter	Resource Foraging
Falcón-Cortés et al. (2019), Nauta et al. (2020a), Nauta et al. (2020b)	Lévy parameter and memory utilization probability	Resource Foraging
de Lope et al. (2015), Lee and Kim (2019), Kazakova et al. (2020), Lee et al. (2020)	Task switching probability threshold	Task Allocation
Viswanathan et al. (2000), Viswanathan et al. (2008), Harikumar et al. (2019), Pang et al. (2019)	Lévy parameter	Target Search

#### 2.2.3 Other Metrics

In addition to spatial and probability based metrics, other metrics have also been used to quantify the level of exploration and exploration carried out by MRS. These metrics are detailed and listed in Table 3. To solve their Best-of-*n* problem Prasetyo et al. (2018), Prasetyo et al. (2019) and De Masi and Ferrante (2020), employed contrarian agents that do not change their opinion of the best site. This use of stubborn agents are a method of measuring and guaranteeing a minimum level of exploration carried out by the system.

**TABLE 3:** List of works using their own developed methods or metrics to quantify the level exploration and exploitation carried out by an MAS.

References	Metric	Task
Prasetyo et al. (2018), Prasetyo et al. (2019), De Masi and Ferrante (2020)	Number of stubborn agents	Best-of-*n* Problem
Hornischer et al. (2020)	Number of unique messages broadcast & proportion of agents broadcasting unique messages	Collective Decision-Making
Kwa et al. (2020a), Kwa et al. (2020b)	Correlation between an agent’s direction of travel and directional bearing to target	Target Tracking
Banfi et al. (2015)	Tracking fairness	Target Tracking
Kwa et al. (2021a), Kwa et al. (2021b)	Engagement ratio	Target Tracking
Yan et al. (2021)	Tracking Fairness and time-based occupancy grid map	Target Tracking

In their collective decision-making task Hornischer et al. (2020), attempted to measure and maximize the diversity of their MRS by counting the total number of unique messages being broadcast, as well as the proportion of agents broadcasting these messages within the system. Using this metric, a small number of unique messages or a small proportion of agents broadcasting a message indicated low levels of diversity.

To measure the exploration and exploitation dynamics of an MRS tracking a moving target Kwa et al. (2020a) and Kwa et al. (2020b) proposed a metric based on an agent’s direction of travel and its bearing to the target. This allowed for an individual agent’s contribution, and hence the system’s overall exploration and exploitation dynamic to be quantified. Kwa et al. (2021a) and Kwa et al. (2021b) later identified that a metric based on an agent’s position relative to a target was unable to function when used in a scenario where multiple targets are to be tracked, such as situations defined under the Cooperative Multi-Robot Observation of Multiple Moving Targets (CMOMMT) framework. This led to the introduction of an *engagement ratio*, thereby allowing quantification of a swarm’s EED in the presence of multiple targets by finding the proportion of agents actively moving towards a target.

In their CMOMMT task Banfi et al. (2015), introduced a metric known as the *tracking fairness* that measures the monitoring effort of the MRS across all targets. Should a target receive more attention from the MRS’s agents, the system would have a high tracking fairness score, indicating over-monitoring of a target or too much exploitation being carried out by the system. Conversely, a low fairness score indicates that the agents are monitoring all the targets evenly and that there is a good balance between exploration and exploitation.

To develop a tracking strategy for the same CMOMMT problem using deep reinforcement learning Yan et al. (2021) used an occupancy grid map (OGM) to characterize the environment. A tracking fairness based metric was then used, comparable to the one proposed by Banfi et al. (2015), as well as an exploration metric based on the latest observation time of all grid cells in the OGM.

## 3 Agent Response Methods

Once a system’s level of exploration and exploitation is able to be quantified, the balance between the two activities can then be controlled. The most common method of adjusting this balance performed by an MAS is by changing an agent’s actions in response to locally measured variations in the environment or information originating from the agent’s neighbors. This method of controlling exploration and exploitation can elicit two types of response from an agent: 1) trigger a discrete change in state of an agent, causing it to perform a predefined set of actions associated to either exploration or exploitation, or 2) cause a small change in an agent’s actions, resulting in a small change in bias of the agent’s actions towards either end of the exploration or exploitation spectrum.

### 3.1 Changes in State

**FIGURE 2:**
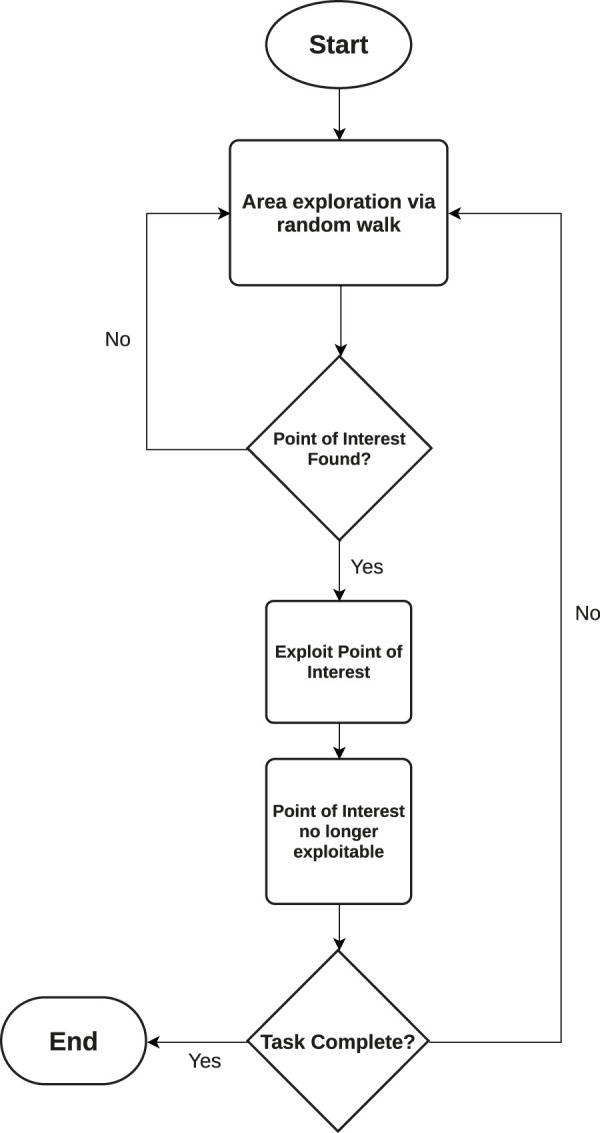
Flowchart detailing how an agent deterministically decides on its response when provoked by environmental stimuli.

When deploying changes in agent states, a large change in an agent’s behavior is effected in response to a given stimulus observed by either an agent, a subset of agents or the entire MAS itself. This gives agents the ability to switch from an exploratory to an exploitative behavior, and vice-versa allows for them to better adapt to their local environment, with the aim to improve the overall performance of the system. These strategies tend to base their exploration strategies on random walks (Zedadra et al., 2017), followed by information exploitation driven by either a metaheuristic algorithm or direct travel towards a point of interest should one be detected. A flowchart of such changes in agent response can be seen in Figure 2.

These changes in response can either be triggered by an agent itself as a reaction to its local environment or a coordinating agent can trigger this response change in a certain subset of the MAS. The latter set of strategies usually involves a centralized controller or locally designated leader to determine which agents are to change their response patterns. As can be expected, each set of strategies comes with its own advantages and disadvantages. For instance, while using decentralized strategies, an MAS is able to retain its flexibility, robustness, and scalability. However, keeping these properties of the system comes at the expense of more precise control over the overall system’s EED. Using a set of strategies involving a centralized controller, an MAS user is able to exert greater and more accurate control over the system’s EED. However, a trade-off must be found, which inevitably impacts one of the system’s three key characteristics of flexibility, scalability, and robustness.

#### 3.1.1 Self-Determined State Changes

In decentralized systems, changes in an agent’s state stem from its own reaction to changes in its local environment. Based on this information, an agent decides for itself if it will carry out exploratory or exploitative actions. When determining their own state in response to their environment, an agent normally takes into account local observations carried out by itself as well as information originating from its neighbors. This allows an agent to react quickly to any changes in its local environment, allowing the MAS to retain its system flexibility. Agents are also not reliant on a leader or central controller assigning it an appropriate response, allowing the system to maintain its scalability and robustness. However, given this decentralized control strategy, the MAS will have less control over the overall amount of exploration and exploitation carried out. A list of such strategies and associated tasks can be found in Table 4.

**TABLE 4:** List of works using self-determined state change strategies to influence a system’s exploration and exploitation dynamics.

References	Strategy	Task
Razali et al. (2010), Razali et al. (2012)	Agent behaviors selected using AIS	Dynamic Shepherding
Hecker et al. (2015)	Random walk search phase & agent assignment exploitation phase	Resource Foraging
Nauta et al. (2020a), Nauta et al. (2020b)	Lévy Walk exploration & memory driven exploitation	Resource Foraging
Zedadra et al. (2018), Zedadra et al. (2019)	Dedicated exploration & exploitation Lévy parameters	Resource Foraging
Li et al. (2014), Zhang et al. (2014)	Random search & PSO tracking strategy	Target Capture
Kolling and Carpin (2006), Kolling and Carpin (2007), Esterle and Lewis (2017), Esterle and Lewis (2020)	Random search & target following strategy	Target Tracking (CMOMMT)
Frasheri et al. (2020)	Pattern search & target following strategy	Target Tracking (CMOMMT)
Kwa et al. (2020a), Kwa et al. (2020b)	Repulsion based exploration & PSO tracking strategy	Target Tracking
Kwa et al. (2020a), Kwa et al. (2020b)	Repulsion based exploration, PSO tracking strategy & adjustable memory length	Target Tracking
Stogiannos et al. (2020)	Agent behaviors selected using AIS	Target Tracking
Harikumar et al. (2019)	Dedicated exploration & localization Lévy parameters	Target Suppression
Lee and Sim (1997), Jun et al. (1999)	Agent behaviors selected using AIS	Task Assignment

##### 3.1.1.1 Dedicated Exploration and Exploitation Strategies

In various systems, agents may switch between pre-programmed exploratory and exploitative behaviors. To capture multiple static targets, the approach proposed by Li et al. (2014) initialized their agents with a random search behavior to explore the environment. In most works involving simulations, a target signal is modeled by using an objective value function, representing the type of information to be exploited by the MAS (e.g., radio signal intensity, chemical plume concentration, etc.). When a target signal was encountered, agents would then employ a PSO algorithm, allowing the system to exploit the target signal and capture the target. After capturing the target, the agents would revert to their original state and resume their random environment search.

Similar changes in state and strategy have been utilized in searching for and tracking dynamic targets. In the tracking of multiple dynamic objects of interest under the CMOMMT framework Esterle and Lewis (2017) and Esterle and Lewis (2020) programmed their agents to initially explore the area randomly. However when an object of interest was detected, it would transition into a tracking mode, change its response, and ultimately alter its velocity to follow that point of interest. The authors were able to demonstrate that this mixed strategy of random motion and following a target yielded better tracking performances than an exploration-only strategy in which the agents only move around randomly.

To track a fast moving target Kwa et al. (2020a), Kwa et al. (2020b), Kwa et al. (2021a) and Kwa et al. (2021b), initially used inter-agent repulsion to promote area exploration. When a target was detected, an agent and its neighbors would activate a PSO-based tracking behavior, allowing the target’s information to be exploited, facilitating target tracking. This behavior would be disabled once the target is no longer detected. The ability to dynamically select between the use of exploratory and exploitative strategies appropriately was demonstrated to be key in facilitating the tracking of the target as it prevented over-exploitation of the target and over-exploration of the search space.

Changes in state may also be triggered by signals originating from neighboring agents. In their work Kolling and Carpin (2006) and Kolling and Carpin (2007), explored the use of so-called “help calls” broadcast from an agent when a target is predicted to be leaving its observation range. Other robots that receive this help call that are not involved in tracking a target move towards the robot requesting help to assist with the tracking task. The use of help calls is also explored by Esterle and Lewis (2017) and Esterle and Lewis (2020) who studied their applications in conjunction with various response and communication models. Frasheri et al. (2020) expanded on this work, incorporating a *willingness to interact* parameter that determined the propensity of an agent to aid in the tracking of a target.

Agents are also able to form dynamic groups dedicated to exploiting information stemming from a point of interest. The formation of groups is more commonly done together by means of a central controller or through a locally designated leader. However, these dynamic groups can still be formed using decentralized decision-making. Zhang et al. (2014) proposed a strategy to search for multiple targets in which agents are initialized in the random-walk search mode. When a target is found, a dynamic group comprised of a maximum of 10 agents is formed. Agents in this group exploit the target’s emitted signal through the use of a PSO algorithm. At each time-step, agents broadcast their measured target signal strengths and individually decide if they should join or leave the group.

The use of changes in agent response is not limited to applications within the domain of target search and tracking. In their foraging task Hecker et al. (2015), split their foraging task into two separate phases. Initially, agents follow a search phase guided by a random walk. After a specified amount of resources has been gathered, agents are assigned to travel to specific regions to exploit high rewarding resource clusters. During this exploitation phase, the agents travel to their allocated locations via a directed random walk. This path becomes straighter, and hence more directed over time, allowing the agents to reduce the amount of exploration carried out.

In a similar foraging task Nauta et al. (2020a) and Nauta et al. (2020b), stored the location of resource patches in a limited agent-based memory fashion. The authors used a truncation probability that determined if an agent’s exploration via Lévy walks are to be cut short. This allowed agents to randomly transition between informed movements and random searches. By increasing the truncation probability, agents of a system could be made to use information from its memory more often, leading to a system-level EED that favors exploitation. Kwa et al. (2021a) and Kwa et al. (2021b) also made use of memory in their target tracking task. However, instead of changing the probability of an agent accessing information from its memory, the system’s EED was tuned by changing the length of an agent’s memory. A system can be made to favor exploitation with the use of longer memory lengths and exploration with the use of shorter memory lengths.

##### 3.1.1.2 Artificial Immune System Strategies

The ability to change between distinct behaviors is also one of the cornerstones of *Artificial Immune System* (AIS) strategies. First proposed by Farmer et al. (1986), AIS was later defined by De Castro et al. (2002) as an “adaptive system, inspired by theoretical immunology and observed immune functions, principles and models, which are applied to problem solving.” Such strategies are commonly used in computational tasks such as computer security, pattern recognition, and optimization (Liu et al., 2006; Dasgupta, 2012), however they have also seen recent applications in various MRS (Raza and Fernandez, 2015). In this family of strategies, all agents are given a repository of behaviors or rules. These behaviors are associated with state values that are influenced by various stimuli in the environment, such as the presence of tasks or targets, as well as by the state values of encountered agents. The behavior with the highest state variable at a given time-step is the one chosen to be carried out.

Initial adaptations of AIS for use in MRS were mostly limited to the domain of task search and allocation. In the initial work done by Lee and Sim (1997); Jun et al. (1999), the repository of behaviors consisted of four strategies: 1) aggregation, 2) random search, 3) dispersion, and 4) homing to carry out a target tracking task. When a robot encounters another robot, each robot re-evaluates its behavioral state values. Subsequently, the behavior with the largest state value is the one chosen to be activated. Using a similar strategy Sun et al. (2001), also demonstrated the AIS’s potential in situations where is a large influx in quantity of tasks to be performed by the system. The same strategy was applied by Razali et al. (2010) and Razali et al. (2012) in a dynamic shepherding scenario, as well as by Stogiannos et al. (2020) in a dynamic target tracking task.

##### 3.1.1.3 Lévy Parameter Changes

Instead of using vastly different movement policies to effect changes in the response of agents, agents using Lévy walks can vary their Lévy parameter. As explained in Section 2.2.2, changing the Lévy parameter *μ* can be used to influence the overall EED of an MAS; higher Lévy parameter values are used to shorten the walk lengths of agents, prioritizing exploitation, while conversely lower *μ* values increase walk lengths and hence favor exploration. As such, several MAS make use of changing their Lévy parameter to influence its EED instead of using dedicated exploration and exploitation algorithms.

For a system carrying out a destructive foraging task Zedadra et al. (2018), Zedadra et al. (2019), used a time-based system paired with a “satisfaction” parameter to determine the transition from the exploitation mode to the exploration mode. When exploring, the MAS used a small Lévy parameter to generate long-step sizes. This was changed to favor smaller steps when a target was found to encourage exploitation. While in the exploitation mode, an agent’s satisfaction parameter value was increased for each time-step a target was encountered and decreased when no target was met. An agent would only transition from the exploitation to exploration when both the satisfaction value reached zero and the minimum time limit for the exploitation mode was reached.

To determine the location of forest fires Harikumar et al. (2019), proposed using a temperature threshold to determine an agent’s state. In their MAS, all agents are initialized in a searching state, using a low *μ* value to direct area exploration. Should an agent detect a temperature higher than a specified threshold value, it would transition to a more exploitation-focused strategy by employing either a directional-driven Brownian search or regular Brownian search strategy, drawing from a Lévy probability distribution function with a higher *μ* value.

#### 3.1.2 Assigned State Changes

While self-determined changes in state are mostly found in fully decentralized systems, assigned state changes can be found in fully centralized systems (i.e., systems where the movement of all agents is dictated by a central controlled) and hierarchical systems (i.e., systems with designated ‘leader’ agents determining the actions of a small proportion of agents). Using assigned state changes, when a stimulus is found, only a specific subset of the system comprised of a fixed number or predefined subset of agents is assigned to exploit the new stimulus information. This is in contrast to the responses seen in the previous section in which agents are able to decide for themselves if they should switch behaviors. Using such behaviors allows for greater and more precise control over an MAS′ EED. However due to the more centralized nature of such strategies, an MAS must sacrifice a certain degree of flexibility, scalability, and robustness. Using this class of strategies, the assignment of roles and responses to agents can either be done dynamically as the task progresses, or during the initialization process. A list of works assigning agent response patterns can be found in Table 5.

**TABLE 5:** List of works using assigned agent responses to influence a system’s exploration and exploitation dynamics.

References	Strategy	Task
Sánchez et al. (2018)	Dynamic sub-swarm membership	Area Exploration
Prasetyo et al. (2018), Prasetyo et al. (2019), De Masi and Ferrante (2020)	Stubborn agents that do not change opinion of site	Best-of-*n* Problem
Thenius et al. (2016), Donati et al. (2017), Hamann (2018), Varughese et al. (2018)	Dedicated environment exploration and long term monitoring (exploitation) agents	Environment Monitoring
Carlisle and Dozier (2000), Hu and Eberhart (2002), Zhan et al. (2009)	Forced re-initialization of a subset of candidate solutions	Optimization
Blackwell and Bentley (2002), Blackwell and Branke (2006), Blackwell (2007)	Dedicated exploratory and exploitative particles	Optimization
Chira et al. (2008), Pintea et al. (2009)	Ant Colony System paired with agents with preset pheromone sensitivity	Optimization (Traveling Salesman Problem)
Couceiro et al. (2011a), Couceiro et al. (2011b), Couceiro et al. (2014), Couceiro and Portugal (2018)	Dynamic sub-swarm membership	Target Search
Wang and Meghjani (2020)	Search strategies assigned based on agent’s distance to target and target-to-searcher ratio	Target Search
Yan et al. (2018)	Forced re-initialization of robots	Target Search
Wang and Cavallaro (2016), Schranz and Andre (2018)	Predetermined static agents for target search and mobile agents for target tracking	Target Tracking (CMOMMT)
Ni and Yang (2011), Cao and Sun (2017), Cao and Sun (2018), Ni et al. (2018), Ge et al. (2018), Farid et al. (2018)	Specified number of agents closest to target chosen to utilize a target tracking strategy	Target Tracking

##### 3.1.2.1 Dynamic Agent Assignment

While using assigned state changes does indeed reduce a system’s flexibility, a certain degree of flexibility can still be retained by having agents assigned to tasks while a mission is being carried out. Ni and Yang (2011) demonstrated a dynamic agent assignment strategy in a 2D environment. In their target search scenario, agents were initialized with a random walk to explore the environment. When a target is found, a leader agent is designated to coordinate a dynamic alliance comprised of four agents. While these agents carry out the target pursuit task, the rest of the agent continue with exploring the environment. Ni et al. (2018) later built on this work and demonstrated this type of strategy for finding targets in an underwater 3D environment. The agents employed initially used a Lévy flight-based cruise phase to search for one of multiple targets. When a target is found, a sub-swarm is formed using a centralized self-organizing map (SOM), which are also used in transfer learning neural networks (Karimpanal and Bouffanais, 2019). Only members of this sub-swarm carry out exploitation of the target’s signal through the use of the Dolphin Swarm Algorithm (DSA) while the rest of the agents continue exploring the search area. In both strategies, after successfully capturing the target, the pursuing sub-swarm’s agents disband and continue with the search process by reverting to the cruise phase. Important to this strategy is the dynamic nature of the sub-swarm’s membership, meaning that agents in the sub-swarm may be substituted should it stray too far from the target.

In their work done on tracking and monitoring of multiple dynamic targets with UAVs in a 3D environment Farid et al. (2018), employed a strategy where all agents carried out a patrol phase based on the Lévy flight. When a target is detected, the agent closest to the target is designated as leader. The leader selects the next two agents that are nearest to the target’s location to form a sub-swam comprising of three agents. Only members of this sub-swarm would transition to a PSO-based tracking mode to exploit this information and pursue the target until the target leaves the surveillance area. The leader is also able to substitute agents within the sub-swarm with other agents should the original agents move too far away from the target.

Similar techniques that assign agents to targets have also been demonstrated by Ge et al. (2018) in aquatic surface operations and by Cao and Sun (2017), Cao and Sun (2018) in underwater operations. Instead of using a random walk, the authors generate a potential field fitness function based on unexplored areas of the environment and the location of obstacles within the operating area. Exploration is carried out through the use of a PSO algorithm that makes use of this potential field by driving agents towards unexplored regions of the environment. When a target is found, the four agents nearest to the target enter a tracking mode and are assigned to pursue that target until it is caught by using the PSO algorithm on the target’s emitted signal. Upon a successful target capture, these agents revert to the search mode to look for more targets. In another study done on search and rescue strategies by Wang and Meghjani (2020), searcher strategies are assigned to an agent based on the agent’s distance to a potential target and the ratio of searchers to targets.

The size of the sub-swarms need not be fixed. In Couceiro et al. (2011a),Couceiro et al. (2011b), Couceiro et al. (2014) and Couceiro and Portugal (2018), work is done on a Robotic Darwinian PSO (RDPSO), adapted for use with ground robots from the original Darwinian PSO by Tillett et al. (2005). Sánchez et al. (2018) later expanded on this work with applications using underwater robots carrying out exploration in a 3D environment. This strategy balances the swarm’s EED through social exclusion or inclusion of agents and is done by splitting the MRS into multiple sub-swarms and independent “wanderers” that search for the solution individually. All of these sub-swarms execute the same target tracking strategy independently and there is no direct information transfer between different sub-swarms. However, there are some system-level rules applied during each iteration that serve to keep the membership of the sub-swarms evolving, thereby facilitating indirect information transfer.

The system level rules that govern this social exclusion or inclusion of the RDPSO algorithm is the “punish-reward” rule, as well as the sub-swarm creation and deletion mechanisms. If a sub-swarm improves its best fitness value, it will add the best performing wanderer from the excluded agents into its sub-swarm membership. However if a sub-swarm has not improved on its fitness value, it will exclude its worst performing agent, which becomes an independent “wanderer” itself. If after a specified period of time, a sub-swarm has been rewarded more times than it has been punished, it is allowed to generate a new sub-swarm. This new sub-swarm will be composed of best performing agents among the wanderers. However, if the sub-swarm has been punished more times than it has been rewarded, its population will be reduced, and upon reaching the minimum number of agents, disbanded and all member agents will be socially excluded. These two mechanisms are key to the dynamic nature of the system’s sub-swarms and provide a method of indirect information transfer between the groups. The exploration carried out by the independent wanderers and the indirect information transfer facilitated by the dynamic properties of the sub-swarms’ membership guarantees a minimum amount of exploration carried out by the overall system.

In numerical optimization, where it is easier to control the movement of individual agents, it may be decided that sub-swarms may not be needed. As such, a common strategy observed in this field is to trigger the re-initialization of particles within the search space (Carlisle and Dozier, 2000; Hu and Eberhart, 2002; Zhan et al., 2009). Using these strategies, a central controller will command the agents to reset their positions to a random location within the search space after a predefined time period or when a large change is detected in the environment. The strategy was adapted for use in robotics by Yan et al. (2018) for leak source detection. In their proposed strategy, the average fitness value of each agent was calculated and a subset of agents that had a fitness value less than the system mean were instructed to restart their search process from a different location.

##### 3.1.2.2 Preassigned Roles

Besides dynamic response assignment, agents can be assigned a response pattern during their initialization. Doing so allows for greater control over the system’s behavior and exploration and exploitation balance in exchange for less system flexibility. In numerical optimization Blackwell and Bentley (2002), Blackwell and Branke (2006), Blackwell (2007), proposed a PSO swarm inspired by the atomic model, consisting of agents clustered close to each other dedicated to solution exploitation and agents surrounding the central cluster dedicated to exploration.

To solve the *Traveling Salesman Problem* (TSP) Chira et al. (2008), Pintea et al. (2009), proposed using an Ant Colony System in conjunction with two sets of agents associated with two different levels of pheromone sensitivity. Agents more sensitive to pheromones are more likely to be influenced by environmental stimuli and are therefore able to exploit information stemming from marked search areas. Conversely, less sensitive agents are more likely to be independent explorers, allowing the discovery of potential search regions. The use of Ant Colony System that requires agents to modify and exploit their operating environment will be elaborated upon further in Section 4.2.

This use of dedicated agents has also seen applications in the particular class of dynamic best-of-*n* problems. In this scenario, an MAS is tasked with coming to a consensus on which option out of *n* possible options offers the best conditions to satisfy the needs of the system (Valentini et al., 2017). Prasetyo et al. (2018), Prasetyo et al. (2019), De Masi and Ferrante (2020) introduced dedicated “stubborn” agents to their system. These agents did not change their opinion of a given site while other agents were allowed to change their opinion based on interactions with other agents using the voter model. These stubborn agents guaranteed a minimum amount of exploration carried out by the MAS and allowed the system to respond more quickly when there were sudden large changes in site quality.

Similar dedicated agents have also been used in various MRS. In the subCULTron project, a heterogeneous swarm of robots was developed and built for the monitoring of an underwater environment (Thenius et al., 2016; Donati et al., 2017; Hamann, 2018; Varughese et al., 2018). This system relied on a more mobile set of agents, the “aFish”, for exploration of areas within the environment, and a less mobile set of agents with higher sensor capabilities, the “aMussel”, to exploit the local environment and collect data over long periods of time.

Similar heterogeneous MRS have been used for tracking of mobile targets in hybrid camera systems consisting of mobile and static cameras (Wang and Cavallaro, 2016; Schranz and Andre, 2018). However, in contrast to the subCULTron MRS, static cameras serve as the dedicated exploratory agents, identifying targets within the coverage area. The mobile cameras are then assigned to pursue the detected targets by a central controller.

### 3.2 Small Response Changes

In contrast to the large changes in state that result in drastic changes in an agent’s behavior and response, an MAS′ agents may choose effect smaller changes in its response patterns. While these minor adjustments in an agent’s behavior may not result in such extreme changes in a system’s overall EED, they allow for more precise adjustments of the level of exploration and exploitation carried out by an MAS. A list of such strategies can be seen in Table 6.

**TABLE 6:** List of works using small response changes to influence the level of exploration and exploitation of an MAS.

References	Strategy	Task
Howard et al. (2002)	Exponential inter-agent repulsion strength	Area Coverage
Vallegra et al. (2018), Zoss et al. (2018)	Exponential inter-agent repulsion & attraction gradient	Area Coverage
Ganganath et al. (2016)	Exponential inter-agent repulsion with “selfishness” term to drive exploration	Area Coverage
Ebert et al. (2020)	Varying agent environment sampling time	Area Characterization
Okumura et al. (2018)	Varying time interval at which robots regroup to trade map information	Area Exploration
Schumer and Steiglitz (1968), Azad and Hasançebi (2014), Hansen and Ostermeier (2001), Hansen (2006)	Adaptive step size	Optimization
Blackwell and Bentley (2002)	Exponential inter-agent repulsion strength	Optimization
Kernbach et al. (2009), Schmickl et al. (2009), Bodi et al. (2012), Hereford (2013), Bodi et al. (2015), Kengyel et al. (2016), Wahby et al. (2019)	Varying agent wait time based on local fitness value	Optimal Area Aggregation
Pang et al. (2019), Nauta et al. (2020c)	Variable Lévy parameter	Resource Foraging
Sardinha et al. (2020b)	Variable Lévy parameter & interruption of Lévy walks	Resource Foraging
Hecker et al. (2015)	Random walk that gradually becomes more directed	Resource Foraging
Jatmiko et al. (2007)	Exponential inter-agent repulsion strength	Target Search
Sutantyo et al. (2013)	Using Lévy walks and Firefly optimization algorithm to generate points of attraction for each agent with different attraction weights	Target Search
Zhang et al. (2019)	SRLA and SRLAMR agent interaction schemes	Target Search
Coquet et al. (2019), Coquet et al. (2021), Kwa et al. (2020a),Kwa et al. (2020b), Kwa et al. (2021a),Kwa et al. (2021b)	Exponential inter-agent repulsion and attraction strength	Target Tracking
Parker and Emmons (1997), Parker (2002), Kolling and Carpin (2006), Kolling and Carpin (2007)	Linear inter-agent repulsion strength & variable target attraction strength	Target Tracking (CMOMMT)

#### 3.2.1 Attraction-Repulsion Dynamics

**FIGURE 3:**
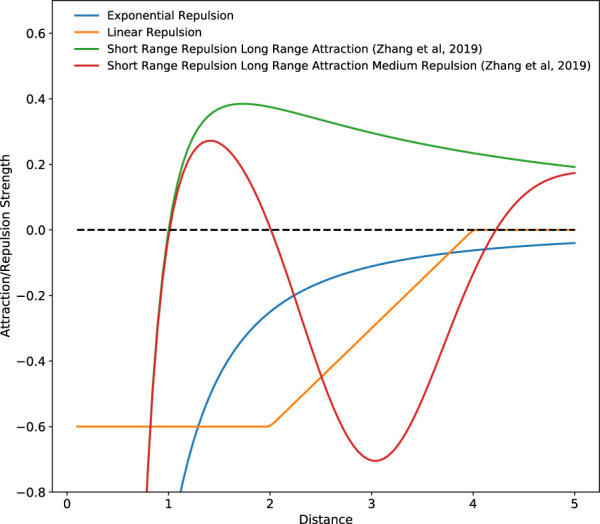
Inter-agent interaction forces generated by various repulsion and attraction schemes. Positive values indicate an attractive force and negative values indicate a repulsive force.

Effecting changes to an agent’s attraction and repulsion strength with respect to its neighbors or specified points in the environment is one of the more common small response changes used. Increasing an agent’s repulsion strength has the effect of augmenting the amount of exploration carried out by that agent. Conversely, increasing an agent’s level of attraction favors more exploitative actions. Done across an entire MAS, this allows for regulation of a system’s EED. When applied within an MRS context, the inter-agent repulsion also generates a collision avoidance behavior as a byproduct of this mechanism. Most repulsion vectors are calculated as follows:

$$V_{i}=\frac{a}{\|x_{i,t}{\|}^{d}}\frac{x_{i,t}}{\left\|x_{i,t}\right\|},$$ (2)

where *a* is the set repulsion parameter, *d* is a constant, and *x*_*i*,*j*_ is the vector from an agent *i* to a point of repulsion *j*. This point of repulsion is usually a neighboring agent and using an equation similar that seen in Eq. 2 results in higher repulsion strengths being generated when two agents are close together. Hence, when using such repulsion equations, agents are strongly repelled from each other when they are immediately next to each other (see Figure 3), discouraging excessive exploitation of the environment.

In a swarming MRS developed to find the source of a dynamic odor plume, Jatmiko et al. (2007), inspired by the Charged PSO algorithm developed by Blackwell and Bentley (2002), increased the strength of an inter-agent repulsion parameter when robots came within a predefined distance of each other to avoid high levels of clustering within too small an area. It should be noted that in the experiments done by Jatmiko et al. (2007), the size of the environment and the number of robots in operation, and hence the global average swarm density, are known *a priori*. This allowed for the authors to tailor the repulsion parameters and optimize the robot’s search behavior accordingly (Hayes et al., 2002). A similar repulsion scheme was adapted by Coquet et al. (2019) for use in their dynamic target tracking swarm. Similarly, to prevent excessive agent clustering while carrying out a target tracking task Kwa et al. (2020a), Kwa et al. (2020b), Kwa et al. (2021a), Kwa et al. (2021b) employed an adaptive repulsion algorithm that gradually increased the strength of inter-agent repulsion while agents were exploring the area. This repulsion strength was also gradually decreased while agents were attempting to move towards a target. In these works, a repulsion equation similar to that seen in Eq. 2 was used.

In addition to inter-agent repulsion to prevent excessive aggregation when tracking multiple mobile targets Parker and Emmons (1997), also varied an agent’s attraction strength to a target based on the distance between an agent and the target, with targets further away triggering a weaker attraction response. Furthermore, the authors also implemented agent-target repulsion when an agent moved too close to a target to prevent excessive exploitation. Developing this strategy further Parker (2002), Kolling and Carpin (2006), Kolling and Carpin (2007) reduced the weight of the point of attraction generated by targets already being tracked, preventing the overlap of robot sensing areas, further reducing the over-exploitation of a target. In these works, a linear repulsion function was used, as seen in Figure 3, instead of the exponential function that is normally used.

In a target search task, Sutantyo et al. (2013) ignored inter-agent repulsion and focused only on the use of attraction by combining the Lévy walk together with a Firefly Optimization algorithm to generate a velocity vector for each agent at each time-step. An agent’s velocity is influenced by the attractiveness value of other agents within its communications range, encouraging exploitation, and a random point within the environment obtained from a Lévy distribution, encouraging exploration. Using the proposed strategy, an agent’s attractiveness is based on a function on the amount of time elapsed since it last encountered a target. An agent that sees a neighbor with a high level of attractiveness will bias its movements more towards its neighbor than towards the point of exploration.

Besides the inter-agent repulsion behavior more commonly utilized Zhang et al. (2019), also implemented an inter-agent short range repulsion and long range attraction (SRLA) component within their strategy based on inter-agent distances, effectively preventing excessive exploration for their *k*-capture game carried out by an MAS. In addition to this, they developed a Short Range Repulsion Long Range Attraction Medium Range Repulsion (SRLAMR) scheme where the inter-agent force transitioned between repulsion and attraction depending on the inter-agent distance. These two attraction and repulsion schemes were derived from the work done by Gazi and Passino (2004), Chen et al. (2011) and can be seen in Figure 3. Comparing these schemes with the exponential repulsion schemes, the authors found that using the exponential repulsion scheme resulted in too much exploration, preventing targets from being captured, while the SRLA and SRLAMR schemes promoted agent clustering, facilitating the capture of targets. However, the SRLAMR scheme outperformed the SRLA scheme due to the former resulting in the dynamic formation and dissolution of agent clusters over time. These clusters were also distributed across the search space, demonstrating a balance between exploration and exploitation, allowing the system to capture fast-moving targets. By combining and adjusting the strength of separate inter-agent attraction and repulsion fields Coquet et al. (2021), has demonstrated that a stable equilibrium position can be attained where agents maintain a fixed relative position to each other even though the entire swarm may be in motion. This allowed for the overall surface of an MRS, and hence the overall EED of the swarm, to be controlled even though the entire system may be moving.

Inter-agent attraction and repulsion is also commonly used in MAS tasked with area coverage and monitoring, which is a task that requires the prioritization of exploration over exploitation. Howard et al. (2002) demonstrated how agents of an MRS can be distributed and deployed across a search space through the use of an exponential repulsion scheme. In addition to inter-agent repulsion, robots were also repelled by obstacles in the environment, allowing robots in their MRS to distribute themselves evenly across complex environment shapes.

This area coverage task was expanded on by Vallegra et al. (2018), Zoss et al. (2018) who demonstrated the use of an physical MRS system for a dynamic area coverage problem: i.e., covering an area that changed shape over time. To achieve this, they augmented inter-agent repulsion together with a potential field gradient attracting agents outside the designated monitoring area towards the area perimeter. This was done to limit unnecessary exploration and encourage exploitation in the correct areas.

Comparable attraction-repulsion dynamics were also demonstrated by Ganganath et al. (2016). In their area coverage strategy inter-agent repulsion was used to prevent coverage area overlap, therefore stopping over-exploitation of the environment. This was supplemented with an agent-based “selfishness” term that directs the agents towards less visited areas, thereby promoting system exploration.

#### 3.2.2 Random Walks

In environments where there is a lack of information, strategies based on attraction-repulsion dynamics may not be viable due to the lack of stimuli to drive agent activity. To operate in such information sparse environments, random walks, such as Lévy walks, are a popular strategy to promote area exploration. However, the use of Lévy walks may result in inefficient searching due to possible inter-robot collisions and coincidental clustering of robots. As such Pang et al. (2019), developed a random walk in which agents determine their walk sizes based on a local swarm density estimation. This allows an agent to carry out longer random walks—and hence carry out exploration when in a less crowded areas and shorter walks—while allowing the agents to carry out more exploitation when in areas with higher agent densities. The use of an adaptive step size is a common class of strategies in computational optimization algorithms (Schumer and Steiglitz, 1968; Hansen and Ostermeier, 2001; Hansen, 2006; Azad and Hasançebi, 2014). In such strategies the step size is varied based on the path taken by the solution; the step size is decreased should the solution stay around the same region of the search space for several iterations, enabling better exploitation, and increased should the solution move in the same direction for several consecutive time-steps, facilitating exploration.

Similarly Nauta et al. (2020c), also varied the Lévy parameter, *μ*, of individual agents using a parameter based on the amount of time elapsed since an agent’s last target encounter. Using this strategy, an agent’s Lévy parameter value continuously decreases towards unity while an agent does not encounter any targets. Doing so allowed for an agent to gradually prioritize exploration if it does not encounter a target for long periods of time. In addition to adjusting an agent’s Lévy parameter based on the number of resource encounters within a given time-frame. Similar to this Sardinha et al. (2020b), also explored varying a parameter that determines an agent’s propensity of prematurely interrupting a Lévy walk should the agent experience a decreasing number of resource encounters. By increasing the chances of interruption, the agent is more likely to change its search path and explore the search area.

During their exploration phase while carrying out a foraging task Hecker et al. (2015), used an informed random walk that is initially undirected and localized to look for patches of resources. As an agent gathers information about resource locations, these walks become more directed as agents tend to move directly towards higher yielding patches. This allows agents to exploit high rewarding resource patches while retaining some ability to explore the environment for better locations.

#### 3.2.3 Time Intervals

The use of environment sampling intervals as well as communication time intervals is also a method that can be used to control the level of exploration and exploitation carried out by an MAS. This is the primary method used by the BEECLUST algorithm first proposed by Kernbach et al. (2009), Schmickl et al. (2009), Bodi et al. (2012). This algorithm was formulated after observing bees tending to cluster in regions of higher temperature and in regions where clusters are already present. When implemented in an MRS, agents attempted to find an area illuminated by the source with the highest light intensity where the light intensity of the sources varied over time. Using this algorithm, agents measure their local environment’s light intensity only when they collide with other agents and proceed to wait in position for a period of time. This waiting time is determined by each agent individually and increases with the measured light intensity. This leads to a positive feedback loop where longer wait times lead to more aggregation in areas with higher light intensity. This phenomenon is known as Motility-Induced phase Separation (MIPS) (Cates and Tailleur, 2015) and eventually results in higher levels of exploitation carried out by the system in areas of high agent density. Algorithms making use of MIPS were later studied by Bodi et al. (2015) for use in underwater environments and demonstrated in physical robot platforms by Kengyel et al. (2016).

While effective, the positive feedback loop caused by MIPS may cause the system to over-exploit a specific area and result in lower levels of responsiveness in the presence of a highly dynamic light source. As such Wahby et al. (2019), modified algorithm to account for an agent’s local swarm density when determining its wait time. This modification allowed an agent to move off earlier when located in an area of high swarm density, preventing over-exploitation and improving the system’s ability to respond and adapt to a highly dynamic environment.

In their area exploration task using agents with very low communication ranges Okumura et al. (2018), varied the time interval at which robots regrouped to trade map information at a predefined position. Using this strategy, shorter time intervals forced agents to regroup more often, favoring exploitation. Conversely, longer intervals allowed agents to move further before regrouping, allowing for more exploration. The simulation results obtained suggested that shorter time intervals and higher levels of exploitation performed better in smaller environments while longer time intervals and more exploration yielded better results in larger environments.

Sampling time intervals were also studied by Ebert et al. (2020) in their area characterization and collective decision-making task. The authors developed an algorithm that caused agents to sample their environment and communicate with their immediate neighbors only after specific time intervals. In changing these observation time intervals, it was shown that shorter intervals result in agents taking more measurements, increasing their rate of exploitation, and being able to accurately characterize their local area. However, the overall system displays lower decision accuracy when characterizing the entire area. As with the work done by Okumura et al. (2018), this is the result of over exploitation done by the agents due to multiple measurements being taken of the same region of the environment, leading to a high level of spatial correlation between the robots’ location and the MRS consensus. In contrast, longer observation intervals lead to less samples being taken. However, since the robots sample over a larger distribution of tiles, a higher level accuracy obtained by the system when attempting to characterize the whole environment.

### 3.3 Area and Task Assignment

In addition to the previously mentioned methods, agents can also be assigned to a specific area or task to manage the overall EED of an MAS. Doing so usually requires an estimation of where points of interest are expected to be and the location of agents at any given point in time. Also, doing so allows for a belief map of to be generated that maps both the estimated demand for and supply of agents. Moving agents from areas of high supply and low demand allows for more exploration to be carried out by an MAS and prevents the over-exploitation of certain areas. These maps can be generated by individual agents themselves based on local measurements or be done on a global level and transmitted down to the agents. A list of such strategies is summarized in Table 7.

**TABLE 7:** List of works using agent area and task assignments to influence the level of exploration and exploitation of an MAS.

References	Strategy	Task
Alvarenga et al. (2020)	Area partitioning	Patrolling
Wu et al. (2020)	Agent deployment probability threshold	Resource Foraging
Jung and Sukhatme (2006)	Agent utility distribution to drive robot movement	Task Allocation
Lee and Kim (2019), Lee et al. (2020)	Belief maps & task transition probability	Task Allocation

Using independently maintained agent supply and demand estimation maps in their target tracking task Jung and Sukhatme (2006), derived a utility distribution to drive robots towards areas of the search space in which they would provide the highest amount of utility to the entire system. Similarly Alvarenga et al. (2020), in their patrolling task used a central controller to partition a search area into multiple sub-regions that were assigned to individual agents. Doing this allowed areas that saw more target visits to be partitioned into smaller sub-regions, thus facilitating the assignment of more agents and higher levels of exploitation in that specific area. Belief maps were also used by Lee and Kim (2019), Lee et al. (2020) in a dynamic task assignment scenario in conjunction with task transition probabilities. Using their strategy, agents would increase their probability of performing tasks with higher demand but low agent supply.

In situations where communications are denied in the operating environment or when belief maps are not used, agents can be activated probabilistically to carry out a task based on their level of experience. Such strategies are usually carried out through the use of probability thresholds, with less experienced agents being more likely to be deployed to carry out their own exploration of the environment. Wu et al. (2020) utilized this type strategy in a resource foraging scenario where agents with less map information had a higher probability of being deployed in the environment to gather resources, giving them the chance to build up their personal maps of the operating environment. While this type of strategy does not yield the best performance, it allows the system to maintain a level of robustness by having a large pool of experienced agents. This in turn enables the system to continue with its task in the event where its most experienced agents are lost.

## 4 Information Dissemination Methods

The second broad category of approaches towards controlling the exploration and exploitation dynamics of an MAS is based on adapting the way information is disseminated across the system. This system-wide information transfer can be achieved by means of direct or indirect communication strategies. Indirect methods are often observed in nature with agents altering the environment to convey information to fellow agents: this process is known as stigmergy. For example, some ant colonies use pheromones to guide worker ants to various food sources (Sumpter, 2010; Perna et al., 2012). Direct methods can be observed in colony of bees when the dancer bee dances to signal the onlooking bees on the quality of the food sources found. With engineered systems, radio transmissions offer a direct means of communication that is the most common among MRS for dissemination of information. These methods are generally used in tandem with an agent response method that controls the amount of information an agent has access to. By doing so, an agent’s actions and responses can be influenced indirectly and can have large effects on the system’s collective response to the environment (Bouffanais, 2016). A list of main strategies that influence an MAS EED through changing the level of communications between agents can be found in Table 8.

**TABLE 8:** List of works using different information dissemination strategies to influence the level of exploration and exploitation of an MAS or MRS.

References	Strategy	Task
Rausch et al. (2019)	Adjusting communications range	Collective Decision Making
Crosscombe and Lawry (2021)	Changing number of communication neighbors	Distributed Consensus
Rahmani et al. (2020)	Changing attention limit of agents	Distributed Consensus
Strömbom (2011)	Changing interaction radius and field of vision angle	Distributed Consensus
Mateo et al. (2017)	Changing interaction radius	Distributed Consensus
Kuan (2018)	Forced switching network model	Distributed Consensus
Janson and Middendorf (2005), De Oca et al. (2009), Liu et al. (2016), Blackwell and Kennedy (2018)	Changing number of communication neighbors	Optimization
Tillett et al. (2005)	Dynamic sub-swarm membership	Optimization
Talamali et al. (2021)	Adjusting communications range	Optimal Size Aggregation
Pitonakova et al. (2016)	Adjusting communications range	Resource Foraging
Kano et al. (2020)	Adjusting communications range based on internal workload state	Task Allocation
Tang et al. (2020a)	Changing an agent’s number of neighbors in network based on fitness value	Target Search
Mateo et al. (2019), Kwa et al. (2020a), Kwa et al. (2020b), Kwa et al. (2021a), Kwa et al. (2021b)	Changing number of communication neighbors	Target Tracking
Esterle and Lewis (2017)	Varying communication link strength and number of communication neighbors	Target Tracking (CMOMMT)

### 4.1 Changes in Network Topology

MAS that use direct communication methods can control the information flow within the system by varying several possible communication network parameters, such as number of neighbors (for individual agents), agent’s communication range, and various techniques like sub-swarm assignment and forced network change. These are further elaborated in the following sub-sections.

#### 4.1.1 Number of Neighbors

One of the network parameters that can affect the dissemination of information is the number of direct (or immediate) neighbors that each agent possesses. It is worth stressing that the concept of “neighbor” shall not be limited to classical definition according to a metric or Euclidean distance. Instead, this concept should be considered in its most general form, which is conveniently captured by the network theoretic concept of node degree (Shang and Bouffanais, 2014). In general, most of the studies consider the number of neighbors as a system-level parameter—i.e., all the agents *i* have the same number of neighbors *k*_*i*_ = *k*—although it is absolutely possible to envisage a heterogeneous system, in which the *k*_*i*_ values are different. When considering this parameter *k*, it is crucial to distinguish between directed and undirected networks. Specifically, with a directed network, the information flows across the system in one direction, i.e., if agent *A* is a neighbor of agent *B* then the information flows from *A* to *B*, but not necessarily the other way round. Directed networks make the relationship between agents asymmetric in terms of information flow. On the other hand, with undirected networks, that relationship is symmetric, meaning that the information flows in both directions when two agents are connected.

Through these repeated local information exchanges, an agent is able to form local decisions based on the information gathered by itself and its immediate neighbors—still in the network sense. For instance, using the PSO algorithm, changing the number of neighbors an agent communicates with is one of the key methods for controlling the level of exploration and exploitation carried out by the system (Janson and Middendorf, 2005; De Oca et al., 2009; Liu et al., 2016; Blackwell and Kennedy, 2018; Kwa et al., 2020a; Kwa et al., 2020b; Kwa et al., 2021a; Kwa et al., 2021b). By increasing *k*, information about the global “best” solution can be propagated more swiftly around the system, thereby encouraging exploitation. Conversely, by reducing the degree *k*, information propagation is slowed down. This prevents agents from over-exploiting the same information, thereby increasing system exploration.

To prevent agents from being trapped in local optima, Tang et al. (2020a) developed a robotic PSO strategy using a synthesized dynamic neighborhood. In this framework, an agent expands its neighborhood should it be determined to be trapped in a local optimum. By using such expanding neighborhoods, trapped agents have a chance to find and follow a “leader” with a better fitness value, thereby progressing towards the global optimum. This concept was further explored in Mateo et al. (2017), Kit et al. (2019), Mateo et al. (2019), Kwa et al. (2020a), Kwa et al. (2020b), Kwa et al. (2021a), Kwa et al. (2020b), where various *k*-nearest neighbor systems were evaluated in multiple targets tracking and multi-robot mapping operations. It was observed that system performance tends to improve as *k* increases to an optimal value (Mateo et al., 2017; Mateo et al., 2019; Kwa et al., 2020a; Kwa et al., 2020b; Kwa et al., 2021a; Kwa et al., 2021b). Beyond this optimum, increasing *k* yields detrimental effects to the system performance due to the MAS’ tendency to over-exploit information at higher levels of connectivity. This optimal level of connectivity was also found to be dependant on the rate at which the environment evolves; within a fast changing dynamic environment, a system with lower *k* performs better due to the slower spread of outdated information across the system, thereby promoting exploratory behavior.

Such observations have also been made when observing MAS in static environments. Crosscombe and Lawry (2021) observed an optimal *k* when attempting to obtain multi-agent consensus in a noisy static environment and reduce the error between this consensus and the ground truth. The simulations also demonstrate a well-known result in network science where efficient and well connected networks result in better short-term results to the detriment of long-term performance while more diverse and less connected networks result in poorer short-term results and better performances in the long-term (Lazer and Friedman, 2007; Judd et al., 2010; Lorenz et al., 2011). When attempting to reach a consensus on the best direction of movement Rahmani et al. (2020), demonstrated that there was an ideal *attention limit*, the number of stimuli an agent can process at any given point in time, at which the accuracy of the system consensus was maximized. This occurs because a large attention span results in strongly connected flocks that tend to converge and exploit a single source of information very quickly while a small attention span leaves the system vulnerable to small variations in an individual’s movement. Due to the rapid convergence on a single source of information—i.e., over-exploitation, it is very difficult for informed individuals to inject new sources of information to the system and promote further exploration, resulting in low system accuracy. However, when agents have a very low attention limit, the system becomes susceptible to noise and variations in the movement of individual agents, causing the system to explore many different potential sources of information, also leading to the degradation of the system’s performance (Mateo et al., 2017).

Kit et al. (2019) also used this method of control by changing the number of neighbors in the network to perform collective mapping operations. In this work, the individual robotic units get access to sensor information based on the *k*-nearest-neighbor network. This information, in the form of occupancy grid maps, is used by the agents to form localized decisions about the next waypoint. This allows for a fully decentralized control of the agents to move towards unexplored spaces via Frontier exploration (Yamauchi, 1997).

#### 4.1.2 Communication Range

For the operation of MRS, the maximum communications range between two robots is of significant interest as it is one of the key factors that determines the maximum number of neighbors in an agent’s communications network. As such, many studies carefully focus their investigations on the limitations associated with the communication range.

Pitonakova et al. (2016) studied the swarming system behavioral plasticity at the collective level in dynamic environments with a class of foraging problems. The nature of the changes occurring in the environment is associated with the quality of food sources in the environment—i.e., at each food patch quality change interval, the deposited quality changes. Various strategies have been explored in these dynamic environments. Some strategies are based on the idea of exploration-exploitation by tuning two parameters: the probability of switching to an exploring agent and the communication range. It is interesting to note that in static environments, a “maximum” information sharing rate is beneficial when the system makes collective decisions. However, when the swarm operates in a dynamic environment, information transfer needs to be carefully controlled to achieve a balance between exploration and exploitation. This balance is essential for the system to achieve high levels of adaptivity to find new and possibly better quality foraging sites, instead of constantly exploiting the same foraging site(s).

Along the same vein Rausch et al. (2019), worked on finding and maintaining a balance between peer agreement (exploitation) and exploration of new solutions. Their simulated swarm is modeled after locusts in a two-dimensional space and their movement is restricted to either clockwise or counter-clockwise. The agents use information available to them (e.g., neighbors and its own information) and taking into account system noise to locally decide whether to move in a clockwise or counter-clockwise direction. This work analyzed the relation between social feedback and noise, and collective coherence. The method aims to minimize the number neighborhood connections while maximizing the system’s collective coherence and response to the sudden addition or removal of agents (i.e., changing swarm sizes). Classically, the global agreement is an emergent behavior resulting from the sum of local coordination efforts. However, if the neighborhood is small, the agents are more willing to adapt their actions to the local environment, a result also demonstrated by Strömbom (2011), Talamali et al. (2021) in a movement consensus task and in a dynamic optimum area aggregation task respectively. However, this over-willingness to adapt leaves an agent vulnerable to random fluctuations of its neighbors’ output—i.e., the swarm becomes undecided, with a low degree of coherence (Czirók and Vicsek, 1999; Buhl et al., 2006). This is similar to using too little neighbors in an agent’s communications network as explained in the previous section. Therefore the strategy deployed controls the swarm by changing the size of its neighborhood, and hence its level of exploration and exploitation.

Kano et al. (2020) proposed a Non-Reciprocal-Interactive-Based (NRIB) model based on the idea of “exploration-exploitation.” Essentially, a non-reciprocal interaction network is directed. Initially, the homogeneous agents are spatially distributed in thefield. Once an agent enters an area of tasks, its workload increases, while the area tasks decreases at a constant rate. As the neighbors detect this increase in workload within its network, they will approach it and execute it cooperatively, effectively exploiting this new source of information. When the tasks are completed, the workload of the robots will naturally decrease and they will start repelling one another, exploring the area and seeking out other tasks.

Similar to the work done by Rausch et al. (2019), the improved NRIB model includes a key parameter to better direct the system’s exploitative activities: the radius of communication range. The intention is for the agents to look for other agents that need help outside of their initial neighborhood. In case where there are too many agents with a high workload, it could create confusion for the agent to determine its next waypoint. Therefore, reducing the radius of communication range can help the agents find the nearest neighbor with the highest workload, allowing exploitative actions to be better directed. An automatic tuning mechanism is implemented to adjust the radius of communication range based on the workload of the neighborhood.

#### 4.1.3 Forced Network Changes

Should a fixed level of connectivity be desired, changes can be made to the network’s structure by changing an agent’s communications neighbors. This allows an agent to maintain the same communication range or number of neighbors, while allowing for more information diversity in the agent’s network. Esterle and Lewis (2017) dealt with online multi-object *k*-coverage. The strategy is inspired by an ant foraging method that consists in both negative and positive feedback loops. When two cameras are connected to each other, this is connection is considered as a link and that will be reinforced as long as the cameras continue to “see” the same object. The strategy also allows for the weakening of this connection as all link strengths decay over time. This decay opens the possibility for it to be used in dynamic environments as the system can unlearn the links, preventing the over-exploitation of outdated information sources.

In the work by Esterle and Lewis (2017), various network structures are used, namely the *k*-nearest, *k*-furthest, and *k*-random networks. The *k*-nearest and *k*-furthest networks are based on Euclidean distance, while the *k*-random network is based on a set of randomly chosen neighbors across the entire system, allowing the communication to gradually expand to “all” cameras. Using topologies such as the *k*-furthest or *k*-random networks essentially allowed the agents to explore a larger area by permitting the gathering of information from neighboring agents further away. This increased amount of exploration was studied while using various response models (e.g., moving to assist in the tracking of the closest target, moving to assist in the tracking of the target with the lowest number of pursuing agents, etc.). Using wider networks paired with the response model increased the level of exploration of an agent as well as allowed the agents to select the most appropriate source of information to exploit that will benefit the system the most.

**FIGURE 4:**
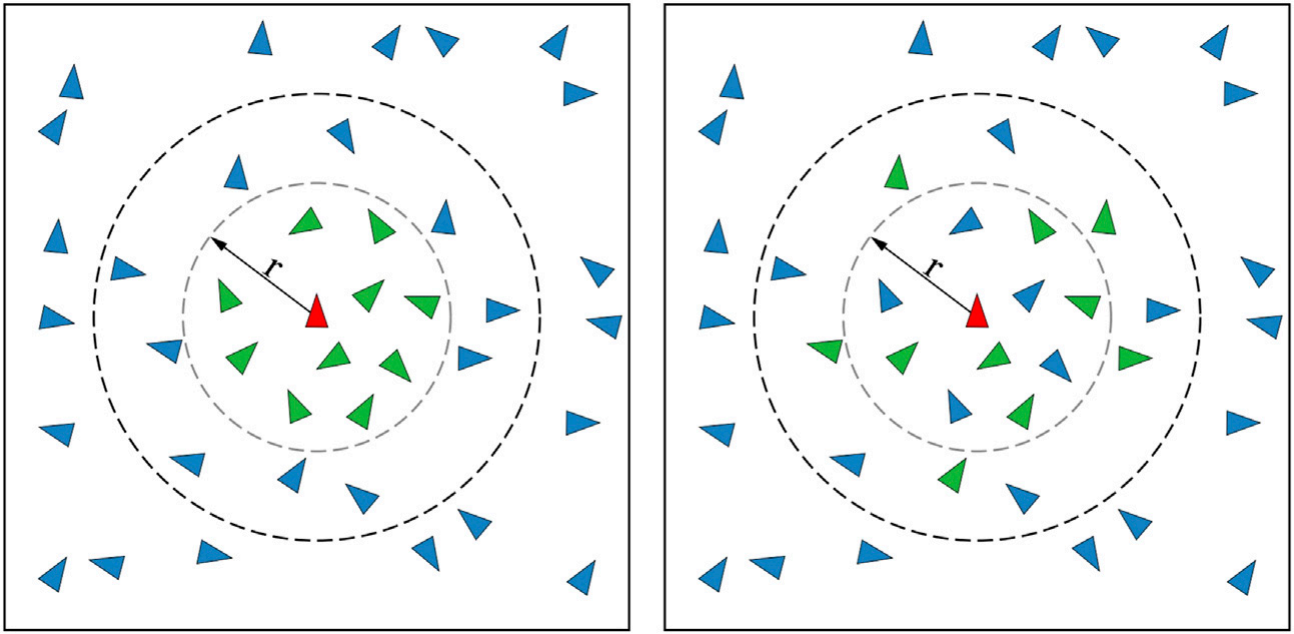
(Left) A standard communication model with an agent in communication with its 10 nearest neighbors. (Right) A forced switching communication model where *k*_*s*_ = 5 of the nearest neighbors are substituted with a more distant set of neighbors within the black circle with a probability of *p*. (Kuan, 2018).

Kuan (2018) demonstrated a “Forced-Switching” network model for their distributed consensus task, where an agent switches its *k*_*s*_ nearest neighbors to a more distant set of neighbors with a given probability, *p* (see Figure 4). This allows agents to obtain a wider, yet still local, information set. Similar to the *k*-furthest and *k*-random network structures used in Esterle and Lewis (2017), this network topology essentially allows an agent to explore the environment more by allowing an agent to sample from a larger set of information. This in turn leads to higher levels of consensus, especially in slow evolving environments.

Lastly, Sekunda et al. (2016) analyzed the influence of switching agents on the topology of the network of interaction when considering simulations of self-propelled particles. The overall coherence of the swarm—measured through its polarization—was found to increase with agent switching.

### 4.2 Stigmergy

Should an MAS operate in an environment where direct agent-to-agent communications are denied, indirect methods, such as stigmergy, can be used. In this framework, an agent is able to elicit a response from other agents in the system by leaving traces in the environment (Theraulaz and Bonabeau, 1999). This concept was first introduced in Grassé (1959) and has been applied in various MAS.

The most notable application of stigmergy is the Ant Colony Optimization (ACO) algorithm, first proposed by Dorigo (1992). It has been used in various optimization tasks ranging from vehicle routing problems to electrical grid management (Mohan and Baskaran, 2012). Using the ACO algorithm, agents deposit markers, also known as pheromones, as they traverse the environment. These pheromones are most commonly used attractively, drawing agents to points of interest, with most commonly used pathways being reinforced with higher pheromone concentrations, hence resulting in a positive feedback loop encouraging exploitation A list of works that influence a system’s EED through the use of stigmergy can be found in Table 9. To prevent over-exploitation and encourage exploration, these pheromones are subject to evaporation, causing the attractive strength of a given pathway to decrease over time (Dorigo et al., 2006). In addition, pheromones can also be subject to diffusion, allowing agents to explore adjacent solutions (Ji et al., 2008; Krynicki et al., 2013). As previously mentioned in Section 3.1.2, Ant Colony algorithms can also be used in systems comprised of two different levels of pheromone sensitivity (Chira et al., 2008; Pintea et al., 2009). This allows for a dedicated sub-group tasked with exploration and another for exploitation.

**TABLE 9:** List of works using stigmergy to influence the level of exploration and exploitation carried out by an MAS.

References	Strategy	Task
Schroeder et al. (2017), Hunt et al. (2019)	Repulsive pheromones	Area Exploration
Dorigo (1992)	Ant Colony Optimization with pheromone evaporation	Optimization
Ji et al. (2008), Krynicki et al. (2013)	Ant Colony Optimization with pheromone diffusion	Optimization
Chira et al. (2008), Pintea et al. (2009)	Ant Colony System with agents of different pheromone sensitivities	Optimization (Traveling Salesman Problem)
Santos et al. (2020)	ACO with solution re-initialization	Path Finding
Herianto et al. (2007), Herianto and Kurabayashi (2009), Johansson and Saffiotti (2009), Khaliq et al. (2014), Khaliq (2018)	Gradient decent with physical data carriers	Target Search
Tang et al. (2019), Tang et al. (2020b)	Gradient decent with vectorial pheromones and physical data carriers	Target Search

In addition to computational optimization tasks, these algorithms have also been implemented in various MRS. Santos et al. (2020) simulated the ACO algorithm’s use in a robot swarm tasked with finding the shortest path between two points in a dynamic environment. However, in addition to the rate of pheromone evaporation, they also re-initialize the system and erases the existing pheromone trail when the system is determined to be approaching stagnation. This strategy essentially triggers a large increase in the amount of exploratory activity carried out by the system’s agents. During this period, the system re-explores the solution domain before it is able to exploit a new set of information gathered through the exploration process and finally converges on a new solution. This is similar to the re-initi alizing of PSO strategies seen previously in Section 3.1.2.

Several groups have worked to bring the ACO algorithm from the virtual to the physical world, showing the feasibility of using such exploration and exploitation control strategies in real-world problems. While using robots to carry out a target search task, (Herianto et al., 2007; Herianto and Kurabayashi, 2009; Johansson and Saffiotti, 2009), spread out RFID data carrier tags around the physical operating space to act as pheromones within the environment. A similar strategy was also employed by (Khaliq et al., 2014; Khaliq, 2018), who used a physical RFID grid to generate a map exploitable by a swarm of robots, guiding them towards a target. Using this type of strategy, robots are equipped with an RFID writer/reader and update the pheromone levels of the tags when they are encountered. Doing so allows an agent to exploit the environmental information by following a gradient descent algorithm towards the target. Similar to their counterparts in computational optimization, these pheromones are also subject to evaporation, preventing the robots from exploiting outdated information. Tang et al. (2019) and Tang et al. (2020b) later built on this work by supplementing the strength of the pheromone with a vector pointing the direction of areas with higher pheromone concentrations. This allows an agent to carry out more effective (targeted) exploitative actions, improving search efficiency and success rates when searching for both dynamic and static targets.

Instead of using pheromones attractively, (Schroeder et al., 2017; Hunt et al., 2019), have also shown that they may be used in a repulsive manner in their area mapping and exploration scenarios. Similar to “no-entry” signals in ant foraging patterns (Robinson et al., 2005), these negative pheromones facilitate area coverage and exploration by preventing agents from taking overlapping paths, thereby encouraging agents to take paths not previously explored. However Hunt et al. (2019), has shown that agent exploration is limited using this strategy when there is an agent located in an area with higher swarm densities. This is because the large amounts of repellent pheromone in the vicinity of an agent limits its movement, preventing it from carrying out any useful exploration.

## 5 Conclusion

The so-called exploration–exploitation dilemma is a common challenge faced by many systems operating on the basis of collective decision-making, including those encountered by various multi-agent and multi-robot systems. This dilemma stems from the fact that, in general, exploration and exploitation tend to be mutually exclusive tasks.

The allocation—be it automated or human driven—of agents to these exploration and exploitation tasks is nontrivial; a large proportion of agents carrying out exploration would indeed result in many points of interest being identified, however, the system would not be able to fully benefit from these points. Conversely, while a large proportion of agents carrying out exploitation would increase the rate at which the system profits from the information currently available, the system would not be able to adapt to a dynamic environment—in particular a rapidly evolving one—and may not be able to maximize the amount it profits from the environment.

Given the challenging nature of this problem and the diverse range of applications for MAS and MRS systems, there is a large body of literature dedicated to studying the balance between exploration and exploitation in such systems. These studies and works often originate from different disciplines (e.g., autonomous multi-agent dynamics, field robotics, and service robotics) and utilize various tools and methods to develop solutions and strategies in relation with their specific problems (e.g., control theory, simulations of self-organized behaviors, and physical experimentation). Given the multidisciplinary nature of this problem and the large body of work reported in the literature over the past decade, in this review, we identify and classify the commonalities among different solutions and frameworks, as well as between the different definitions of exploration and exploitation.

To this end, we have categorized the methods used to control the level of exploration and exploitation carried out by an MAS into two broad categories, namely “agent response methods” and “information dissemination methods.” The latter can be further broken down into “network topology methods”, where a system’s communications network parameters are changed to regulate the flow of information across the MAS, and stigmergy, where an agent is able to indirectly elicit a response from other agents in the system by leaving behind a signal in the environment. Similarly, the broad category of “agent response methods” can be further broken down into: 1) methods that cause an agent to display large and drastic changes in the way it deals with the environment, 2) methods that result in smaller changes in an agent’s response to its environment, and 3) the assignment of agents to areas of interest or tasks found in the environment.

In addition, we have also summarized the methods used in various studies to quantify the level of exploration and exploitation carried out by an MAS. Such quantitative analyses rest upon the proper definition of metrics, which are key to estimating an MAS’ exploration–exploitation balance, thereby allowing one to see if a proposed strategy is working as intended, and make adjustments as needed. However, as is apparent in this review, metrics tend to be closely tied to their specific applications/problems and there is no generalized metric available to measure exploration and exploitation. This important point has also been raised in previous reviews, and a similar observation was also made in reviews about swarm robotics (Brambilla et al., 2013) as well as human-swarm interaction (Kolling et al., 2016). The lack of a single/absolute metric applicable to different problems limits our ability to truly rank different approaches and strategies. Even if there is no guarantee that such a universal metric exists, we strongly believe that the community working on these problems would benefit from ways to compare and rank current MAS strategies.

On top of the metric issue just discussed, it is worth stressing that the fact that most methods/strategies are developed for a specific problem is also an issue. The effectiveness of any approach should clearly be scenario independent. A swarm’s flexibility and adaptivity—i.e., swarm intelligence—is the key concept underpinning this, and it has never been understood as limited to one particular task. In our view, a true swarm should exhibit flexibility and adaptability in vastly different scenarios. Although, seeking swarm intelligence in its most general aspect is a laudable objective, it is still an extremely challenging task in practice, and multi-agent reinforcement learning (MARL) is attempting to do just that (Kouzehgar et al., 2020; Leonardos and Piliouras, 2021). Nonetheless, the design of a benchmark problem would offer the possibility to quantitatively compare the various approaches considered for this problem of exploration–exploitation balance. In the process of designing such a benchmark problem, one can also identify a range of scenarios where swarm intelligence is advantageous for the task. In other words, it is critical to determine the set of environmental parameters that calls for the deployment of a swarm, and for swarm intelligence to enable the effective cooperation of the MAS. For instance, it could be debated if swarm intelligence would really be necessary when dealing with a static problem.

We argue that to probe and quantify swarm intelligence, an MAS has to be subjected to challenging circumstances, which would be equivalent to some sort of “stress test” for flexibility and adaptivity. It is only then can one assess and comment on the level of swarm intelligence demonstrated by a system. We propose that such a “stress test” could take the form a benchmark problem with a range of specific features. As previously explained, the environment in which the MAS is operating should be dynamic and preferably with a relatively fast rate of evolution when compared to the dynamics of a single agent. Specifically, a single-agent system should be unable to cope with the pace of changes in the environment, whereas an MAS made of several of these agents could cooperate to navigate such fast-evolving circumstances by almost continuously adapting its exploration–exploitation balance. The system’s exploratory behavior can be evaluated as it attempts to find or accomplish multiple objectives (e.g., tracking of multiple targets, mapping of multiple areas). The system’s exploitative behavior can be evaluated as it attempts to exploit multiple spatial objectives—i.e., objectives with spatially distributed tasks within the environment. Indeed, a single spatial objective can conveniently be tackled with reduced exploration since the exploitation is highly localized within the environment. From the perspective of a virtual MAS, it is possible to consider ideal sensors (without noise), localization, and inter-agent communication. This therefore eliminates a number of unknowns as the MAS algorithm is being evaluated on a selected benchmark problem, allowing for a more accurate evaluation of the algorithm’s true flexibility.

In this review, we deliberately avoided making a distinction between theoretical analysis, simulations, and actual robotic experiments. However, it is important stressing that performing large-scale experiments with a large number of robots still remain a challenging and complex task (Francesca and Birattari, 2016). While an ideal robotic system will act in the same way as its simulated counterpart, various factors (e.g., robot kinematics, environmental conditions, and noise) will affect the final behavior of an MRS. The differences between simulated and physical systems will only grow as the number of MRS agents increase. Currently, a large proportion of experimental results available tend to be limited in size, scope, and robustness. This highlights the need for more extensive experimentation involving larger systems operating in more complex environments (Hasselmann et al., 2021).

Obviously, this particular area of research is a fast-moving one, given the ever increasing technological readiness level of MRS. As detailed in two recent comprehensive reviews (Dorigo et al., 2021; Schranz et al., 2021), one can expect to see a growth in the number of large-scale MRS deployed to tackle a number of tasks requiring a fine balance between exploration and exploitation, which has been shown throughout this review to be rather complex and challenging.

## Data Availability

The original contributions presented in the study are included in the article/Supplementary Files, further inquiries can be directed to the corresponding authors.
